# Specific convulsions and brain damage in children hospitalized for Omicron BA.5 infection: an observational study using two cohorts

**DOI:** 10.1007/s12519-024-00808-z

**Published:** 2024-05-07

**Authors:** Yuan-Yuan Pei, Hong-Li Wang, Gen-Quan Yin, Yi Xu, Jian-Hao Tan, Xin-Hua Liang, Hui-Ying Wu, Xun-Tao Yin, Chun-Xiao Fang, Jun-Zheng Peng, Zhi-Yuan Wu, Yi Sun, Run Dang, Yu-Feng Liang, Hong-Mei Tang, You-Yi Li, Zhong-Xiang Qiao, Zhi-Cheng Liang, Jian-Ping Tang, Fan-Sen Zeng, Ke-Lu Zheng, Yi-Ru Zeng, Xiao-Jun Cao, Hui-Min Xia, Jian-Rui Wei, Jin-Ling Tang, Si-Tang Gong

**Affiliations:** 1 Clinical Data Center, Guangzhou Women and Children’s Medical Center, Guangzhou Medical University, Guangzhou, 510623 China; 2 Department of Gastroenterology, Guangzhou Women and Children’s Medical Center, Guangzhou Medical University, Guangzhou, 510623 China; 3 Department of Internal Medicine, Guangzhou Women and Children’s Medical Center, Guangzhou Medical University, Guangzhou, 510623 China; 4 Department of Infectious Diseases, Guangzhou Women and Children’s Medical Center, Guangzhou Medical University, Guangzhou, 510623 China; 5 Information Department, Guangzhou Women and Children’s Medical Center, Guangzhou Medical University, Guangzhou, 510623 China; 6 School of Pediatrics, Guangzhou Medical University, Guangzhou, 510623 China; 7 Imaging Department, Guangzhou Women and Children’s Medical Center, Guangzhou Medical University, Guangzhou, 510623 China; 8 Respiratory Department, Guangzhou Women and Children’s Medical Center, Guangzhou Medical University, Guangzhou, 510623 China; 9 Pediatric Intensive Care Unit, Guangzhou Women and Children’s Medical Center, Guangzhou Medical University, Guangzhou, 510623 China; 10 Neonatal Intensive Care Unit, Guangzhou Women and Children’s Medical Center, Guangzhou Medical University, Guangzhou, 510623 China; 11 Rehabilitation Department, Guangzhou Women and Children’s Medical Center, Guangzhou Medical University, Guangzhou, 510623 China; 12 General Pediatrics, Guangzhou Women and Children’s Medical Center, Guangzhou Medical University, Guangzhou, 510623 China; 13 Emergency Department, Guangzhou Women and Children’s Medical Center, Guangzhou Medical University, Guangzhou, 510623 China; 14 Department of Neurology, Guangzhou Women and Children’s Medical Center, Guangzhou Medical University, Guangzhou, 510623 China; 15 Key Laboratory of Structural Birth Defects Research in Guangdong Province, Guangzhou Women and Children’s Medical Center, Guangzhou Medical University, Guangzhou, 510623 China; 16 Cardiovascular Department, Guangzhou Women and Children’s Medical Center, Guangzhou Medical University, Guangzhou, 510623 China; 17 Shenzhen Institute of Advanced Technology, Chinese Academy of Sciences, Shenzhen, 518055 China

**Keywords:** Body temperature, Brain damage, Children, Convulsion, Omicron BA.5, Vaccination

## Abstract

**Background:**

SARS-CoV-2 continues to mutate over time, and reports on children infected with Omicron BA.5 are limited. We aimed to analyze the specific symptoms of Omicron-infected children and to improve patient care.

**Methods:**

We selected 315 consecutively hospitalized children with Omicron BA.5 and 16,744 non-Omicron-infected febrile children visiting the fever clinic at our hospital between December 8 and 30, 2022. Specific convulsions and body temperatures were compared between the two cohorts. We analyzed potential associations between convulsions and vaccination, and additionally evaluated the brain damage among severe Omicron-infected children.

**Results:**

Convulsion rates (97.5% vs. 4.3%, $P$ < 0.001) and frequencies (median: 2.0 vs. 1.6, $P$ < 0.001) significantly differed between Omicron-infected and non-Omicron-infected febrile children. The body temperatures of Omicron-infected children were significantly higher during convulsions than when they were not convulsing and those of non-Omicron-infected febrile children during convulsions (median: 39.5 vs. 38.2 and 38.6 °C, both $P$ < 0.001). In the three Omicron-subgroups, the temperature during convulsions was proportional to the percentage of patients and significantly differed ($P$ < 0.001), while not in the three non-Omicron-subgroups $(P$ = 0.244). The convulsion frequency was lower in the 55 vaccinated children compared to the 260 non-vaccinated children (average: 1.8 vs. 2.1, $P$ < 0.001). The vaccination dose and convulsion frequency in Omicron-infected children were significantly correlated ($P$ < 0.001). Fifteen of the 112 severe Omicron cases had brain damage.

**Conclusions:**

Omicron-infected children experience higher body temperatures and frequencies during convulsions than those of non-Omicron-infected febrile children. We additionally found evidence of brain damage caused by infection with omicron BA.5. Vaccination and prompt fever reduction may relieve symptoms.

**Graphical abstract:**

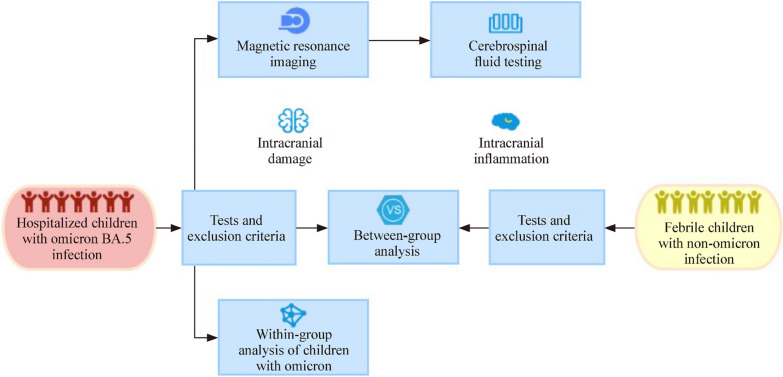

**Supplementary Information:**

The online version contains supplementary material available at 10.1007/s12519-024-00808-z.

## Introduction

Common symptoms in children with severe acute respiratory syndrome coronavirus 2 (SARS-CoV-2) infection include fever, cough, sore throat, fatigue, vomiting, and diarrhea [1, 2]. Previous studies have reported that children infected with early variants of SARS-CoV-2 present with symptoms that differ from those observed in adults [3]. SARS-CoV-2 continues to mutate over time [4–7], and reports on children infected with Omicron BA.5 are limited.

On December 7, 2022, the Chinese government relaxed its dynamic zero-coronavirus disease 2019 (COVID-19) policy, and the country experienced the fastest-increasing wave of Omicron infections globally [8]. The Guangzhou Women and Children's Medical Center is the only hospital the Guangzhou Municipal Government designated to treat children with severe SARS-CoV-2 infection. Previous studies have revealed that febrile convulsions in children occurred in < 7% of cases, with the majority occurring in those < 5 years [9, 10]. However, we observed that the majority of hospitalized children infected with Omicron displayed convulsion signs with cyanosis (blue lips), eyes rolling up and staring, and trembling limbs that did not respond to calls, suggesting that the central nervous system (CNS) of these children may be involved.

Tetsuhara et al. reported severe and repetitive convulsions in a neonate infected with SARS-CoV-2 Omicron [11]. Chongqing University Three Gorges Hospital reported that 84 children infected with Omicron were hospitalized for convulsions between December 11, 2022 and December 26, 2022, similar to our observations [12]. Recently, high rates of convulsions in Omicron-infected children have also been reported [13, 14]. However, the above studies lacked comparative analyses with non-Omicron-infected children.

We aim to evaluate the specific convulsions and body temperature in Omicron-infected children using two cohorts of children—those with and without Omicron fever, analyze the effects of age and vaccination on convulsions, and assess the evidence of brain damage in Omicron-infected children. We also attempted to improve the management of children infected with Omicron based on our findings.

## Methods

### Design and participants

**Fig. 1:**
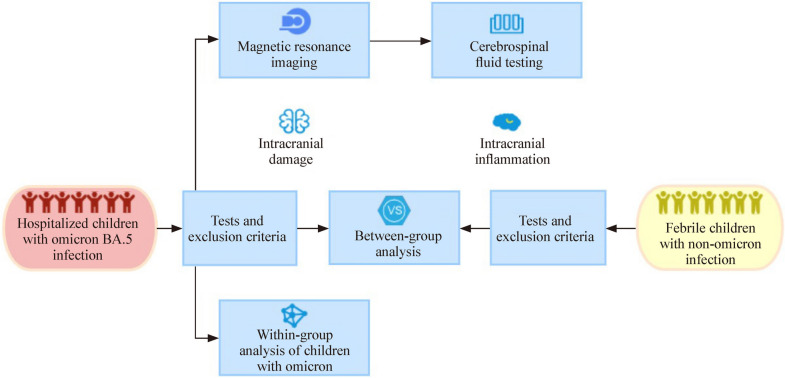
Study flowchart

This study was approved by the Ethics Committee of the Guangzhou Women and Children’s Medical Center ([2023] NO.015A01). Participants included 333 consecutively hospitalized children with SARS-CoV-2 infection and 17,499 non-SARS-CoV-2-infected febrile children attending the fever clinic at the Guangzhou Women and Children’s Medical Center between December 8 and 30, 2022. SARS-CoV-2 infection was confirmed by rapid reverse transcription polymerase chain reaction (RT-PCR). All COVID-19 cases were infected with Omicron BA.5, according to the Municipal Centre for Disease Control and Prevention, which conducted surveillance of the outbreak’s variants using whole-genome sequencing (WGS). Figure 1 illustrates the study flow.

### Measurements and instruments

Epidemiological information, including patient vaccinations, medical history, irrational medicine use before hospital admission, and treatment regimens, was collected through standard epidemiological questionnaires and interviews. The frequency and duration of the convulsions were also recorded. Clinical records and laboratory test results were reviewed from electronic medical records. The data retrieved included blood gas electrolytes, routine blood tests, biochemistry, coagulation, respiratory pathology for RT-PCR, and immunoglobulin M (IgM) antibody testing for acute respiratory tract infections.

Ten respiratory pathogens were detected using RT-PCR, including influenza virus types A and B (INFA and IFVB), respiratory syncytial virus (RSV), enterovirus, *Mycoplasma pneumoniae* (MP), *Chlamydia pneumoniae* (CP), adenovirus (ADV), metapneumovirus, human bocavirus (HBoV), rhinovirus (RhV), and parainfluenza viruses (PIVs). IgM antibodies for nine acute respiratory tract infection pathogens, including *Legionella pneumophila* type 1, MP, rickettsia, CP, ADV, RSV, INFA, IFVB, and PIV types 1, 2, and 3, were tested using indirect immunofluorescence. *Legionella pneumophila* type 1, MP, CP, ADV, and RSV overlapped with the ten pathogens screened using RT-PCR.

Intracranial damage was confirmed via magnetic resonance imaging (MRI) of the brain (Philips Magnetic Resonance Ingenia 3.0 T). Cerebrospinal fluid (CSF) analysis (using CX41 Olympus and BC-7500 CS Mindray), including glucose, chloride, alanine aminotransferase, aspartate aminotransferase, lactate dehydrogenase, and creatine kinase, was performed to diagnose intracranial inflammation.

The patient's temperature on admission was taken via axillary measurement. During an episode of convulsion, the temperature was taken more than once using an electronic forehead thermometer. Finger-clip oximeters were used to obtain functional oxygen saturation readings (SpO_2_). The highest temperature and lowest SpO_2_ recorded were used for analyses in this study.

### Exclusion criteria

**Fig. 2:**
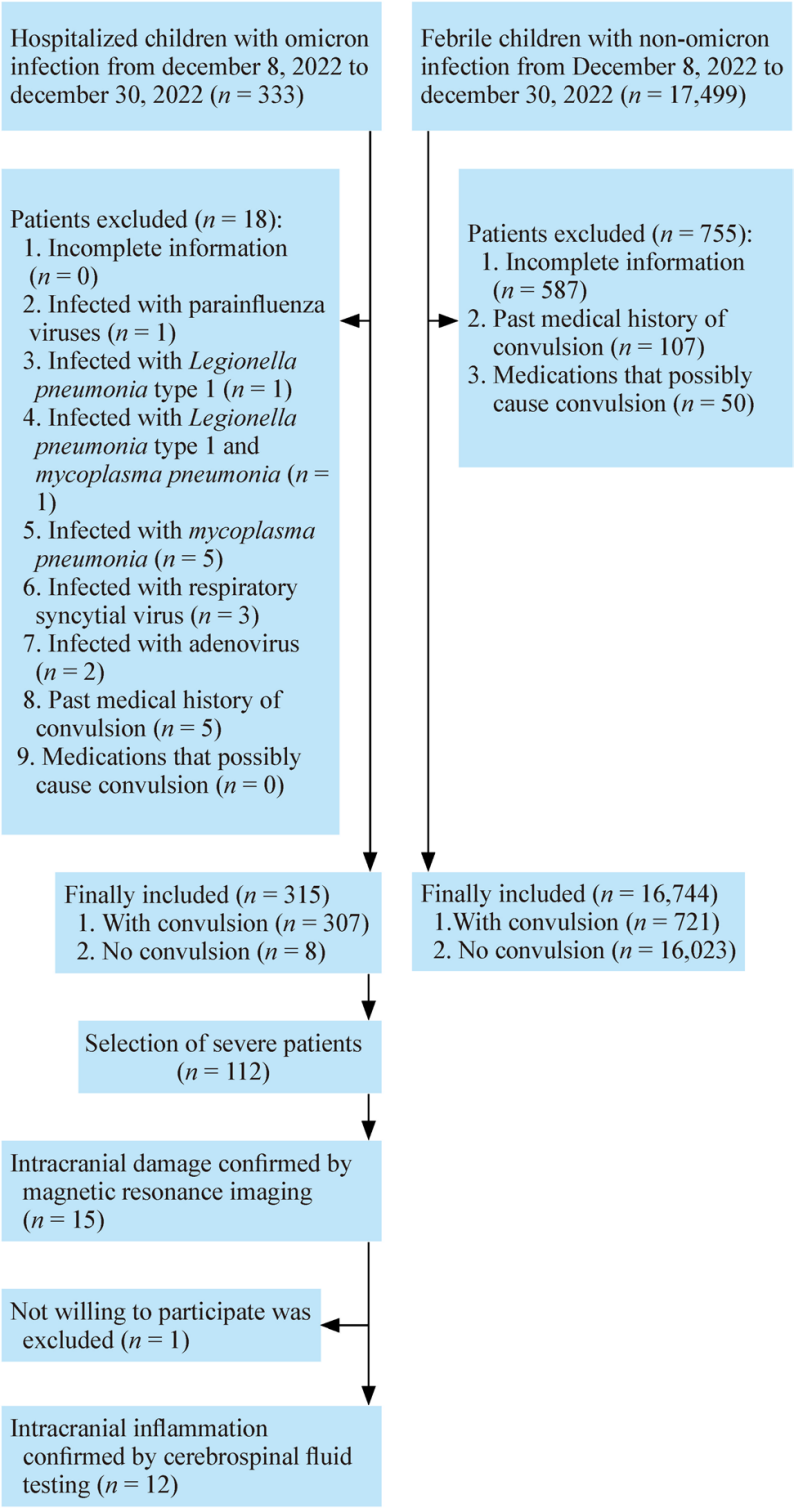
The initial sample and exclusion criteria

An infectious disease physician, a respiratory physician responsible for patient care, and a medical information engineer jointly examined and confirmed the accuracy of the clinical data. In the Omicron-infected group, cases with simultaneous infections with other microbiological agents were excluded. In the group of febrile children with non-Omicron infection, non-infectious diseases, including those caused by diseases such as tumors, autoimmune diseases, and blood disorders, were excluded, leaving 17,499 cases. Additionally, Omicron and non-Omicron cases with convulsions possibly caused by medications or previous diseases were excluded (Fig. 2).

### Statistical analysis

The convulsion rate was compared between Omicron and non-Omicron febrile cases using Pearson’s *X*^*2*^ test. The body temperatures and convulsion frequencies of Omicron and non-Omicron febrile cases were compared using the Mann–Whitney *U* test. The percentage of patients stratified according to body temperature within both groups was compared using Pearson’s *Χ*^*2*^ test. Vaccination rates were compared between Omicron cases with and without convulsions using Fisher's exact test. The non-parametric Spearman's rank correlation coefficient was conducted in Omicron-infected children with convulsions to assess the effect of age and COVID-19 vaccination dose on convulsion frequency, because the non-normality of these data distributions was assessed beforehand using the Kolmogorov–Smirnov test. We considered $P$ ≤ 0.05 significant. Data were analyzed using SPSS version 17 (IBM Corp., Chicago, IL, USA).

## Results

### Characteristics of hospitalized children with Omicron BA.5 infection

The final data included 315 Omicron BA.5-infected children and 16,744 febrile children without Omicron infection after exclusion (Fig. 2). The median age was 2.08 years (0.08–14.00 years) in Omicron-infected children and 2.52 years (0.01–18.00 years) in non-Omicron-infected febrile children. There were 122 (36.6%) and 7454 (42.6%) females in the two groups, respectively. The epidemiological characteristics, clinical features, laboratory test results, and radiological findings of the 315 hospitalized children with Omicron infections are summarized in Table 1. Notably, 260 (82.5%) of these hospitalized children (*n* = 315) were not vaccinated. The epidemiological characteristics and clinical features of 16,744 febrile children with non-Omicron infection were presented in the Supplementary Information (Supplementary Table 1).

**Table 1:** Epidemiological characteristics, clinical features, laboratory tests, and radiologic findings of children hospitalized with Omicron BA.5 infection

Characteristics	*n*/*N*	Percentage or median (range)
*Age*		
Median (range)	315	2.08 (0.08 to14.00)
0–6 month	19/315	6.0%
6 month to 3 year	243/315	77.1%
3–14 year	53/315	16.8%
*Sex*		
Female	114/315	36.2%
Male	201/315	63.8%
*Vaccination history*^a^		
None	260/315	82.5%
One dose	19/315	6.0%
Two doses	30/315	9.5%
Three doses (booster)	6/315	1.9%
RT-PCR testing positive for SARS-CoV-2	315/315	100.0%
*Symptoms at admission*		
Cough	203/315	64.4%
Pharyngeal erythema	125/315	39.7%
Vomiting	121/315	38.4%
Diarrhea	111/315	35.2%
Patients with convulsions	307/315	97.5%
*Number of convulsions per patient during hospitalization*^a^		
1	157/315	49.8%
2–4	132/315	41.9%
≥ 5	18/315	5.7%
No convulsions	8/315	2.5%
*Total convulsion time during hospitalization*^a^		
< 1 min	56/307	18.2%
≥ 1 and < 3 min	57/307	18.6%
≥ 3 and < 5 min	48/307	15.6%
≥ 5 and < 15 min	98/307	31.9%
≥ 15 and < 30 min	36/307	11.7%
≥ 30 and < 60 min	7/307	2.2%
≥ 60 min	5/307	1.6%
*Body temperature*		
> 37.3 °C at admission	311/315	98.7%
Median maximum body temperature in patients with convulsions when not in convulsions °C (range)	307	38.2 (36.8 to 40.5)
Median maximum body temperature in patients with convulsions during convulsions °C (range)	307	39.4 (37.3 to 42.0)
MRI suggesting presence of damage in the brain	15/112	13.4%
CSF analyses suggesting presence of inflammation^b^	12/14	85.7%
*DR or CT on chest suggesting presence of infection in the lungs*	125/196	63.8%
Pneumonia	55/196	28.1%
Bronchitis	4/196	2.0%
Bronchial pneumonia	66/196	33.7%
Abnormal blood routine^b^	221/315	70.2%
Abnormal blood biochemistry^b^	141/315	44.8%
Abnormal coagulation function^b^	161/202	79.7%
*Level of care*		
Level I	15/315	4.8%
Level II	298/315	94.6%
PICU	2/315	0.6%

Data were collected during hospitalization unless specified otherwise

*DR* digital radiography, *CT* computed tomography, *CSF* cerebrospinal fluid, *MRI* magnetic resonance imaging, *PICU* pediatric intensive care unit, *°C* degrees Celsius

^a^Percentages may not add to exactly 100% because of rounding

^b^If any of the tested items are abnormal, it is considered abnormal

### Comparison of symptoms in Omicron-infected children and the control group

**Fig. 3:**
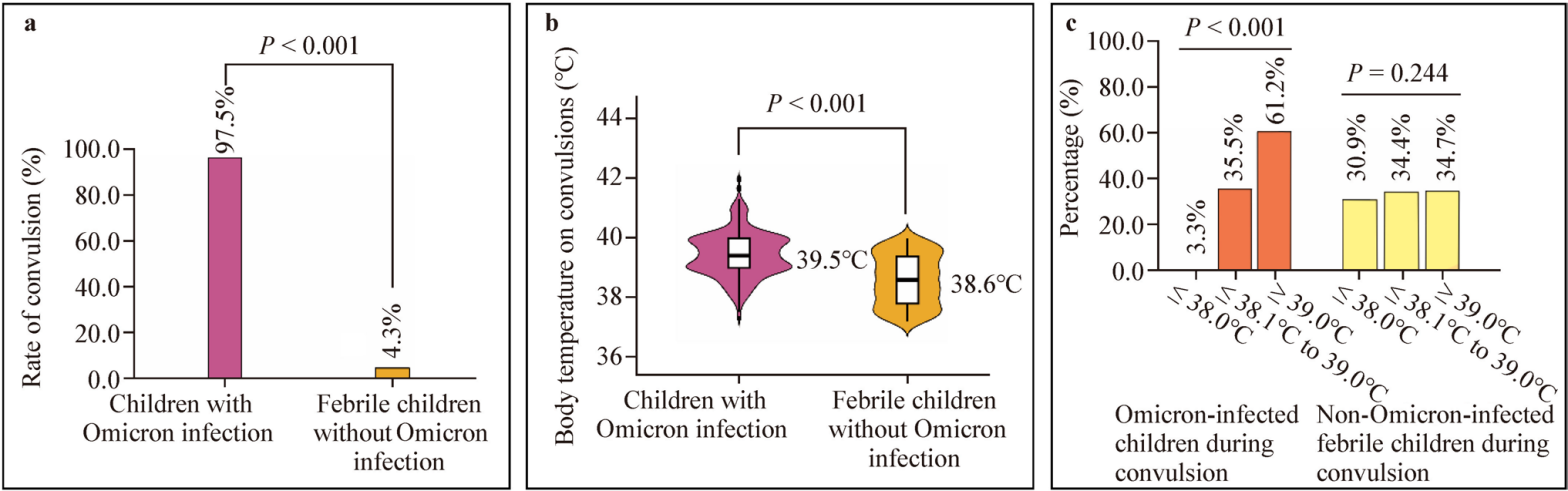
Comparative analysis between groups of the Omicron-infected children and non-Omicron-infected febrile children. **a** Comparison of convulsion rates;** b** Comparison of body temperature during convulsions;** c** Distribution of body temperatures during convulsions in Omicron-infected children and control children. *°C* degrees Celsius

Three hundred and seven (97.5%) of the 315 children with Omicron infection convulsed during hospitalization, which is 22.7 times more than that (4.3% or 721/16,744) in febrile children without Omicron attending the hospital’s fever clinic during the same period (Fig. 3a). The number of convulsions was significantly higher in Omicron-infected children with convulsions than in non-Omicron-infected children with convulsions (median: 2.0 vs. 1.6, $P$ < 0.001). Figure 3b illustrates that the body temperatures of Omicron-infected children were significantly higher than those of non-Omicron-infected febrile children (median body temperature: 39.5 vs. 38.6 °C, $P$ < 0.001). Furthermore, we stratified the temperature data during convulsions for both cohorts and then compared the percentage of patients within groups. Specifically, 3.3%, 35.5%, and 61.2% of the Omicron-infected children had temperatures of ≤ 38.0, 38.1–39.0, and > 39.0 °C during convulsions ($P$ < 0.001), while for the three groups of non-Omicron-infected febrile children, the percentages were 30.9%, 34.4%, and 34.7% ($P$ = 0.244), respectively (Fig. 3c). These findings suggest that higher temperatures in Omicron-infected children may partly be responsible for their higher rates of convulsion compared to non-Omicron-infected febrile children during convulsions.

Pathogen testing was additionally done after SARS-CoV-2 testing of pharyngeal and nasal swabs from the Omicron-infected and control groups. In the group of hospitalized children infected with Omicron, nine positive RT-PCR tests were detected in 117 cases, and four positive IgM antibody tests were found in 77 cases. In the febrile group of non-Omicron-infected children, 6832 RT-PCR tests were positive in 3431 cases, and 673 IgM antibody tests found 289 positive cases. The pathogens with high positivity rates were, in descending order, MP, RSV, ADV, PIV, HBoV, and RhV.

### Within-group analyses of Omicron-infected children

**Fig. 4:**
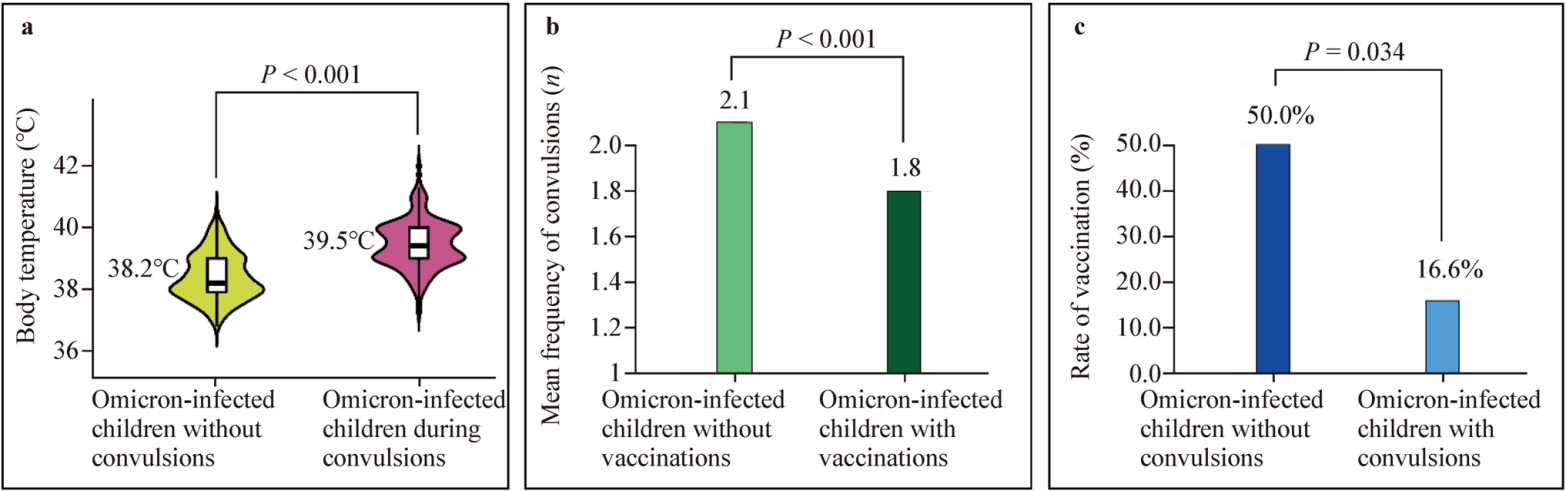
Within-group comparative analysis of children hospitalized with Omicron infection. **a** Comparison of body temperature in Omicron-infected children with and without convulsions; **b** Comparison of the frequency of convulsions in vaccinated and non-vaccinated children infected with Omicron; **c** Comparison of the rate of vaccination in convulsed and non-convulsed Omicron-infected children. *°C* degrees Celsius

The Mann–Whitney *U* test revealed that the body temperatures of 307 Omicron-infected children were significantly higher during convulsions than when they were not convulsing (median body temperature: 39.5 vs. 38.2 °C, $P$ < 0.001) (Fig. 4a). Among the 315 Omicron-infected children, the Mann–Whitney *U* test showed that 55 vaccinated children had a significantly lower frequency of convulsions than 260 unvaccinated children (mean frequency of convulsions: 1.8 vs. 2.1, $P$ < 0.001) (Fig. 4b). The vaccination rate was higher among the eight Omicron-infected children without convulsions than in the 307 Omicron-infected children with convulsions (Fisher's exact test; 50.0% vs. 16.6%; $P$ = 0.034) (Fig. 4c).

A multifactorial correlation analysis of 307 Omicron-infected children with convulsions revealed that the convulsion frequency was significantly correlated with vaccination doses using Spearman's rank correlation coefficients ($P$ < 0.001) (Table 2). Age was negatively but not significantly correlated with the frequency of convulsions (correlation coefficient =  − 0.024, $P$ = 0.681); older children had relatively fewer frequencies of convulsions (Table 2).

**Table 2:** Correlation analysis of convulsions in children with Omicron infection

Variables		Frequency of convulsions	Age	Vaccination doses
Frequency of convulsions	Correlation coefficient	1		
	Significance (bilateral)			
Age	Correlation coefficient	− 0.024	1	
	Significance (bilateral)	0.681		
Vaccination doses	Correlation coefficient	− 0.258	0.559	1
	Significance (bilateral)	0.000*	0.000*	

Correlation analysis of the frequency of convulsions with age and COVID-19 vaccination doses in 307 Omicron-infected children with convulsions using non-parametric Spearman's rank correlation coefficient

*The correlation is significant at a confidence level (double test) of 0.001

### Examination of brain damage

**Fig. 5:**
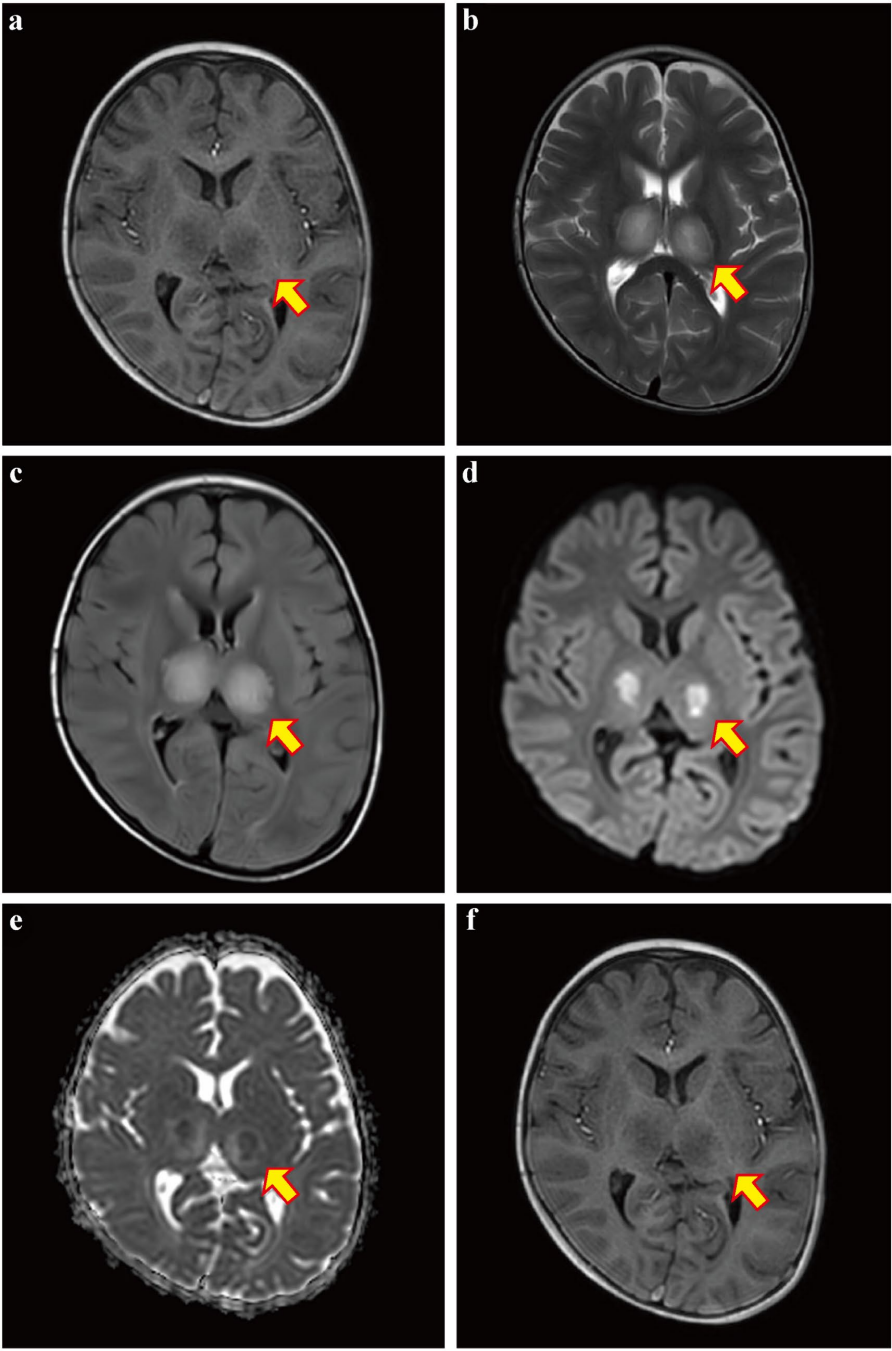
MRI images of a child with Omicron BA.5 infection, showing damage to the thalamus. **a** T1WI showed a low signal; **b** T2WI showed a high signal; **c** FLAIR water suppression showed a significantly high signal; **d** DWI showed a significantly high signal; **e** ADC showed a slightly high signal at the lesion's periphery and a low signal at the center; **f** DWI showed a slightly increased signal in the splenium of the superior corpus callosum. *MRI* magnetic resonance imaging, *FLAIR* fluid attenuated inversion recovery*, ADC* apparent diffusion coefficient*, DWI* diffusion weight imaging

Brain MRI was performed in 112 Omicron-infected children with suspected CNS involvement with peak respiratory frequency > 30 breaths/minute, SpO_2_ < 94% at rest, and > 2 convulsions among 307 children infected with Omicron and presenting with convulsions. Fifteen of them showed brain damage, with major locations in the thalamus, corpus callosum, frontal lobe, parietal lobe, occipital lobe, and basal ganglia. Figure 5 depicts a patient with thalamic damage. Out of these 15 patients, 14 agreed to undergo biochemical analysis, and 12 exhibited signs of intracranial inflammation. In addition, we reviewed the clinical records and found no vaccination records among these 15 children with brain damage. Among 721 children with fever and convulsions who were non-Omicron-infected, 49 patients with complicated convulsions and suspected brain damage underwent brain examination by MRI or computed tomography (CT), and 43 of them also underwent CSF analysis, which found imaging brain damage in 11 cases and intracranial inflammation in three cases, respectively.

WGS of 848 cases sampled in the city by the Guangzhou Center for Disease Control and Prevention showed that all were infected with Omicron BA.5. We performed CSF analysis in the pediatric patients mentioned above to diagnose intracranial inflammation for rapid symptomatic treatment. Due to limited medical resources at the time, no further viral testing of the CSF was performed.

### Outcomes of patients

For the 315 children with Omicron infection, palliative treatments were administered immediately upon admission—two critically ill patients among them were admitted to the intensive care unit (ICU). Initial treatments mainly included ibuprofen or acetaminophen for fever, oseltamivir for viral infections, and cefazolin sodium for bacterial infections. Nebulizing cough and sputum-clearing treatments were administered. During convulsions, intravenous midazolam at a dose of 0.2–0.3 mg/kg was administered to sedate children who convulsed for over 5 min. Furthermore, children with high-grade fever were wiped with wet towels to reduce the fever. Patients with severe disease were also administered 400 mg/kg of gamma globulin to nourish the nervous system and boost immunity for 3–5 days.

By December 30, 2022, 255 (81.0%) of the 315 patients had been discharged because they were significantly better, had stable vital signs, and had normal temperatures for more than 24 h. Out of the 12 children diagnosed with intracranial inflammation, 10 were discharged as the lesions had disappeared or shrunk on MRI reexamination. Additionally, three of these children developed motor dysfunction that recovered after approximately 2 weeks of rehabilitation. One of the two children who remained in the hospital had improved imaging features and had been discharged from the ICU, but was still being treated for motor deconditioning. The other child remained in the ICU. On December 28, 2023, we reviewed the follow-up records of these two patients with intracranial inflammation. One of them had improved, but not fully recovered, motor skills accompanied by right lower extremity claudication. Sadly, the other patient died on January 4, 2023, in the ICU.

## Discussion

This study had several crucial points. First, symptoms of convulsions and fever were comprehensively analyzed based on real-world data from 315 hospitalized children with Omicron infection and 16,744 non-Omicron-infected febrile children during the same period at our hospital. The rate of convulsions in children infected with Omicron was 22.7 times higher than that in non-Omicron-infected febrile children. The body temperatures of Omicron-infected children during convulsions were significantly higher than when not convulsing and the temperatures of non-Omicron-infected febrile children (both $P$ < 0.05). In the Omicron-subgroup, the temperature during convulsions was proportional to the percentage of patients and significantly differed ($P$ < 0.001), while it did not differ significantly in the non-Omicron-subgroup ($P$ = 0.244). These results suggest that prompt fever reduction may reduce the rate of convulsions in Omicron-infected children. Second, there was a significant correlation between the vaccination dose and the frequency of convulsions ($P$ < 0.05), and whether the vaccine was received or not was significantly correlated with the rate of convulsions ($P$ < 0.05). Third, potential evidence of brain damage was observed in severe cases of Omicron infection, and among these affected children, those with brain damage had no COVID-19 vaccination records. Finally, the epidemiological characteristics, clinical features, laboratory tests, and radiological findings of children hospitalized with Omicron BA.5 infection were properly summarized. Overall, the results of this study may serve as a useful reference to facilitate clinical workflows and to develop vaccination policies for Omicron-infected children.

A recent study suggested that Omicron subvariants could be changing how it attacks the human body—infecting respiratory systems and targeting the brain [15]. However, it is important to note that the study used animals instead of humans. Another report suggested that convulsions in children may be related to brain damage [16]. Here, we found that among 112 patients with severe infection, 15 (13.4%) showed intracranial damage in MRI, and 12 (10.7%) had intracranial inflammation detected through CSF biochemical tests. Our clinical evidence suggests that close attention needs to be paid to brain damage in the management of children infected with Omicron BA.5. In addition, the long-term outcomes of intracranial damage or inflammation in a subset of children infected with Omicron are unknown; therefore, continued follow-up of these children by clinicians is required.

Studying the differences in the effects of SARS-CoV-2 on humans as it continues to mutate over time is an important step in clinical management. In a meta-analysis by Misra et al., including 350 studies of 145,721 COVID-19 patients of all ages, up to a third of those patients experienced at least one neurological symptom. That study covered 31 December 2019 to 15 December 2020, which was about 1 year before the Omicron wave. However, it was clear from the paper that convulsions had rarely been reported in children with COVID-19 [17]. Another pre-Omicron wave study reported convulsions in 0.5% of COVID-19 children [18]. In contrast, Sahin et al. reported that seizures were more common during the Omicron period compared to the pre-Omicron (nonvariant, Alpha, and Delta) period [19]. In the early stages of the Omicron (B.1.1.529) wave, 31% of hospitalized children in South Africa developed convulsions [20]. The Tokyo Metropolitan Children's Medical Center in Japan and the pediatric department in Örebro, Sweden, also reported convulsions in children who were later tested for Omicron infection [21, 22]. In the Asia University Hospital in Korea, 62.9% of 97 children who developed convulsions during the Omicron wave were COVID-19 positive, with a significantly higher convulsion rate compared to that of the pre-Omicron wave (0.5% vs. 62.9%, $P$ < 0.001), and patients had a higher mean age and peak body temperature [13]. Thongsing et al. reported increased convulsion rates in infected children (88%, 14/16) during the Omicron wave [14]. A study in Chongqing, China, reported that 84 children hospitalized for the Omicron infection during the Omicron outbreak experienced convulsions [12]. In this study, we observed specific symptoms in a larger group of children infected with Omicron BA.5, found evidence of brain damage in severe patients, investigated strategies to prevent infection in children, and explored palliative treatment approaches.

Our study revealed that both vaccination status and the dosage of the vaccine were significantly associated with disease severity. In addition, these children with brain damage had not received the COVID-19 vaccine. Another study reported that vaccinating children aged 6 months to 5 years reduced the severity of their condition [23]. The Pediatric Pulmonology Department of a University Hospital in Paris observed that during the Omicron wave (between December 15, 2021, and February 28, 2022), the monthly hospitalization rate of unvaccinated children was six times higher than that of fully vaccinated children [24]. A multicenter study conducted in 14 states of the USA reported significant differences in hospitalization rates between vaccinated and unvaccinated children and adolescents [25]. The Centers for Disease Control and Prevention (CDC) in America has expanded the updated COVID-19 vaccines to include children aged 6 months to 5 years [26]. Since January 8, 2023, the Chinese government has implemented "Class B management measures for novel coronavirus infections" [8], implying that more children may be infected with SARS-CoV-2 variants. In this observational study, 83.1% (262) of children hospitalized for Omicron BA.5 infection were under 3 years of age. Currently, children under 3 years are ineligible for vaccination in China. Therefore, if the COVID-19 vaccine is administered to children aged 6 months to 5 years in China, it may potentially reduce the disease severity.

Our study has several limitations. First, the sample size of hospitalized children with Omicron infections was insufficient. Second, owing to the sudden relaxation of epidemic control measures, a short-term surge occurred in hospitalized children with Omicron infection, and many clinicians were also infected. Therefore, the clinical records might be incomplete. In particular, the vast majority of non-Omicron-infected febrile children had mild symptoms and were not hospitalized, and their clinical information was incomplete. Third, pathogen testing, brain MRIs, and CSF analysis were performed at our hospital only when clinically necessary; therefore, not all patients had these tests performed.

In conclusion, our findings demonstrate that children infected with Omicron BA.5 have higher body temperatures than those with common fevers, experience specific convulsions, and may result in brain damage. Vaccination and prompt fever reduction may reduce the disease severity. Further pathophysiologic studies are needed to elucidate the potential effects of Omicron BA.5 on the CNS.

## Supplementary Information

Below is the link to the electronic supplementary material.

Supplementary file1 (DOCX 18 kb)

## Data Availability

Any qualified investigator access to the data for this study will be possible upon request by contacting the corresponding authors and approval by the Ethics Committee of the Guangzhou Women and Children's Medical Center.

## References

[1] Iacobucci G. Covid-19: runny nose, headache, and fatigue are commonest symptoms of omicron, early data show. BMJ. 2021;375:n3103. 34916215 10.1136/bmj.n3103

[2] LaRovere KL, Riggs BJ, Poussaint TY, Young CC, Newhams MM, Maamari M, et al. Neurologic involvement in children and adolescents hospitalized in the United States for COVID-19 or multisystem inflammatory syndrome. JAMA Neurol. 2021;78:536–47. 33666649 10.1001/jamaneurol.2021.0504 PMC7936352

[3] Xu Y, Li X, Zhu B, Liang H, Fang C, Gong Y, et al. Characteristics of pediatric SARS-CoV-2 infection and potential evidence for persistent fecal viral shedding. Nat Med. 2020;26:502–5. 32284613 10.1038/s41591-020-0817-4 PMC7095102

[4] Callaway E. What Omicron’s BA.4 and BA.5 variants mean for the pandemic. Nature. 2022;606:848–9. 35750920 10.1038/d41586-022-01730-y

[5] Imai M, Ito M, Kiso M, Yamayoshi S, Uraki R, Fukushi S, et al. Efficacy of antiviral agents against omicron subvariants BQ.1.1 and XBB. N Engl J Med. 2023;388:89–91. 36476720 10.1056/NEJMc2214302 PMC9749618

[6] Stein SR, Ramelli SC, Grazioli A, Chung JY, Singh M, Yinda CK, et al. SARS-CoV-2 infection and persistence in the human body and brain at autopsy. Nature. 2022;612:758–63. 36517603 10.1038/s41586-022-05542-y PMC9749650

[7] Lin T-F, Zhao ZY, Yang ZR, Li BL, Wei C, Li FX, et al. Hospital strain and Covid-19 fatality: analysis of English nationwide surveillance data. China CDC Wkly. 2022;4:1176–80. 36779170 10.46234/ccdcw2022.236 PMC9906047

[8] Circular on further optimizing the implementation of measures to prevent and control the COVID-19 outbreak, 2022. http://www.gov.cn/xinwen/2022-12/07/content_5730443.htm. Accessed 23 February 2023.

[9] Leung AK, Hon KL, Leung TN. Febrile seizures: an overview. Drugs Context. 2018;7:212536. 30038660 10.7573/dic.212536 PMC6052913

[10] Smith DK, Sadler KP, Benedum M. Febrile Seizures: risks, evaluation, and prognosis. Am Fam Physician. 2019;99:445–50. 30932454

[11] Tetsuhara K, Akamine S, Matsubara Y, Fujii S, Kashimada W, Marutani K, et al. Severe encephalopathy associated with SARS-CoV-2 Omicron BA.1 variant infection in a neonate. Brain Dev. 2022;44:743–7. 35835638 10.1016/j.braindev.2022.06.010 PMC9273474

[12] Liu L, Ou R, Tan Z-Y, Wang N, Chen H. Clinical Analysis of Febrile Seizures in Children with COVID-19 in the Omicron Era: a Single-Center Retrospective Observational Study. Research Square. 2923. https://www.researchsquare.com/article/rs-2758990/v1. Accessed 23 June 2023.

[13] Joung J, Yang H, Choi YJ, Lee J, Ko Y. The impact of omicron wave on pediatric febrile seizure. J Korean Med Sci. 2023;38:e18. 36647218 10.3346/jkms.2023.38.e18 PMC9842486

[14] Thongsing A, Eizadkhah D, Fields C, Ballaban-Gil K. Provoked seizures and status epilepticus in a pediatric population with COVID-19 disease. Epilepsia. 2022;63:e86–91. 35532892 10.1111/epi.17293 PMC9347776

[15] Stewart R, Ellis SA, Yan K, Dumenil T, Tang B, Nguyen W, et al. Omicron BA.5 infects human brain organoids and is neuroinvasive and lethal in K18-hACE2 mice. Biorxiv. 2022. 10.1101/2022.12.22.521696.35677069

[16] Verity CM, Ross EM, Golding J. Outcome of childhood status epilepticus and lengthy febrile convulsions: findings of national cohort study. BMJ. 1993;307:225–8. 8369681 10.1136/bmj.307.6898.225 PMC1678165

[17] Misra S, Kolappa K, Prasad M, Radhakrishnan D, Thakur KT, Solomon T, et al. Frequency of neurologic manifestations in COVID-19: a systematic review and meta-analysis. Neurology. 2021;97:e2269–81. 34635561 10.1212/WNL.0000000000012930 PMC8665434

[18] Cadet K, Boegner J, Ceneviva GD, Thomas NJ, Krawiec C. Evaluation of febrile seizure diagnoses associated with COVID-19. J Child Neurol. 2022;37:410–5. 35286175 10.1177/08830738221086863 PMC9086105

[19] Sahin A, Karadag-Oncel E, Buyuksen O, Ekemen-Keles Y, Ustundag G, Elvan-Tuz A, et al. The diversity in the clinical features of children hospitalized with COVID-19 during the nonvariant, Alpha (B.1.1.7), Delta (B.1.6.172), and Omicron (B.1.1.529) variant periods of SARS CoV-2: caution for neurological symptoms in Omicron variant. J Med Virol. 2023;95:e28628. 36856142 10.1002/jmv.28628

[20] Cloete J, Kruger A, Masha M, du Plessis NM, Mawela D, Tshukudu M, et al. Paediatric hospitalisations due to COVID-19 during the first SARS-CoV-2 omicron (B.1.1.529) variant wave in South Africa: a multicentre observational study. Lancet Child Adolesc Health. 2022;6:294–302. 35189083 10.1016/S2352-4642(22)00027-X PMC8856663

[21] Iio K, Hagiwara Y, Saito O, Ishida Y, Horikoshi Y. Seizure in children with severe acute respiratory syndrome coronavirus 2 Omicron variant infection. Pediatr Int. 2022;64: e15255. 35972066 10.1111/ped.15255 PMC9537988

[22] Ludvigsson JF. Convulsions in children with COVID-19 during the Omicron wave. Acta Paediatr. 2022;111:1023–6. 35098577 10.1111/apa.16276 PMC9303202

[23] Fleming-Dutra KE, Wallace M, Moulia DL, Twentyman E, Roper LE, Hall E, et al. Interim recommendations of the advisory committee on immunization practices for use of Moderna and Pfizer-BioNTech COVID-19 vaccines in children aged 6 months-5 years—United States, June 2022. MMWR Morb Mortal Wkly Rep. 2022;71:859–68. 35771731 10.15585/mmwr.mm7126e2

[24] Taytard J, Prevost B, Schnuriger A, Aubertin G, Berdah L, Bitton L, et al. SARS-CoV-2 B.1.1.529 (Omicron) variant causes an unprecedented surge in children hospitalizations and distinct clinical presentation compared to the SARS-CoV-2 B.1.617.2 (Delta) variant. Front Pediatr. 2022;10:932170. 35832582 10.3389/fped.2022.932170 PMC9271577

[25] Marks KJ, Whitaker M, Anglin O, Milucky J, Patel K, Pham H, et al. Hospitalizations of children and adolescents with laboratory-confirmed COVID-19—COVID-NET, 14 States, July 2021-January 2022. MMWR Morb Mortal Wkly Rep. 2022;71:271–8. 35176003 10.15585/mmwr.mm7107e4 PMC8853476

[26] CDC expands updated COVID-19 vaccines to include children ages 6 months through 5 years, 2022. https://www.cdc.gov/media/releases/2022/s1209-covid-vaccine.html. Accessed 23 February 2023.

