# Supplementary material for: Specific convulsions and brain damage in children hospitalized for Omicron BA.5 infection: an observational study using two cohorts
Source: World J Pediatr. 2024 May 7;20(10):1079–89. doi: 10.1007/s12519-024-00808-z (PMC11502579; doi:10.1007/s12519-024-00808-z)
Supplement: Supplementary file 1 — Supplementary file1 (DOCX 18 kb) [file 12519_2024_808_MOESM1_ESM.docx]

**Supplementary Information**

**Supplementary Table 1.** Epidemiological characteristics, clinical features, laboratory tests, and radiologic findings of non-Omicron-infected febrile children

| **Characteristics** | ***n/N*** | **Percentage or Median (range)** |
| --- | --- | --- |
| **Age** |  |  |
| Median (range) | 16744 | 2.49 (0.08 to 14.00) |
| 0 to 6 mon | 887/16744 | 5.3% |
| 6 mon to 3 years | 11653/16744 | 69.6% |
| 3 to 14 years | 4204/16744 | 25.1% |
| **Sex** |  |  |
| Female | 7181/16744 | 42.9% |
| Male | 9563/16744 | 57.1% |
| **RT-PCR testing positive for**  **SARS-CoV-2** | 0/16744 | 0.0% |
| **Symptoms at fever clinic visit** |  |  |
| Cough | 7179/16744 | 42.8% |
| Pharyngeal erythema | 4052/16744 | 24.2% |
| Vomiting | 2227/16744 | 13.3% |
| Diarrhea | 1963/16744 | 11.7% |
| **Patients with convulsions (frequency)^a^** | 721/16744 | 4.3% |
| 1 | 488/16744 | 2.9% |
| 2 to 4 | 225/16744 | 1.3% |
| ≥ 5 | 8/16744 | 0.0% |
| No convulsions | 16023/16744 | 95.7% |
| **Body temperature** |  |  |
| **At fever clinic visit** > 37.3 ℃ | 15455/16744 | 92.3% |
| Median maximum body temperature in patients with convulsions during convulsions °C (range) | 721 | 38.6 (37.2 to 40.8) |
| MRI or CT suggesting presence of damages in the brain | 11/49 | 22.4% |
| **CSF analyses suggesting presence of inflammation^b^** | 3/43 | 7.0% |
| **DR or CT on chest suggesting presence of infection in the lungs** | 200/437 | 45.8% |
| Pneumonia | 136/436 | 31.2% |
| Bronchitis | 3/436 | 1.0% |
| Bronchial pneumonia | 61/436 | 14.0% |
| **Abnormal blood routine^b^** | 4147/13189 | 31.4% |
| **Abnormal blood biochemistry^b^** | 148/631 | 23.5% |
| **Abnormal coagulation function^b^** | 289/402 | 71.9% |
| **Level of care^a^** |  |  |
| Level I | 50/16744 | 0.2% |
| Level II | 167/16744 | 1.0% |
| PICU | 10/16744 | 0.0% |

Notes: Data were collected at fever clinic visit unless specified otherwise.

^a^ Percentages may not add to exactly 100% because of rounding.

^b^ If any of the tested items are abnormal, it is considered abnormal.

*N* total number of patients or patients tested, *n* number of patients with the condition or event, *DR* digital radiography, *CT* computed tomography, *CSF* cerebrospinal fluid, *MRI* magnetic resonance imaging, *PICU* pediatric intensive care unit, *°C* degrees Celsius
